# Effects of Forest Fragmentation on Connectivity and Genetic Diversity in an Endemic and an Invasive Rodent in Northwestern Madagascar

**DOI:** 10.3390/genes14071451

**Published:** 2023-07-15

**Authors:** Malcolm S. Ramsay, Gabriele M. Sgarlata, Christopher D. Barratt, Jordi Salmona, Bertrand Andriatsitohaina, Frederik Kiene, Sophie Manzi, Miarisoa L. Ramilison, Romule Rakotondravony, Lounès Chikhi, Shawn M. Lehman, Ute Radespiel

**Affiliations:** 1 Department of Anthropology, University of Toronto, Toronto, ON M5S 2S2, Canada; 2 Institute of Zoology, University of Veterinary Medicine Hannover, Foundation, 30559 Hannover, Germany; 3 Instituto Gulbenkian de Ciência, 2780-156 Oeiras, Portugal; 4 German Centre for Integrative Biodiversity Research (iDiv) Halle-Jena-Leipzig, 04103 Leipzig, Germany; 5 CNRS-UPS-IRD, UMR5174, Laboratoire Évolution & Diversité Biologique, Université Paul Sabatier, 31062 Toulouse, France; 6 Planet Madagascar, Antananarivo 101, Madagascar; 7 Faculté des Sciences, de Technologies et de l’Environnement, Université de Mahajanga, Mahajanga 401, Madagascar; 8 Department of Primate Behavior and Ecology, Central Washington University, Ellensburg, WA 98926, USA

**Keywords:** dispersal, connectivity, movement, conservation genomics, Madagascar, habitat loss and fragmentation, rodents

## Abstract

Habitat loss and fragmentation are of concern to conservation biologists worldwide. However, not all organisms are affected equally by these processes; thus, it is important to study the effects of living in fragmented habitats on species that differ in lifestyle and habitat requirements. In this study, we examined the dispersal and connectivity patterns of rodents, one endemic (*Eliurus myoxinus*) and one invasive (*Rattus rattus*), in two landscapes containing forest fragments and adjacent continuous forest patches in northwestern Madagascar. We generated genetic (RADseq) data for 66 *E. myoxinus* and 81 *R. rattus* individuals to evaluate differences in genetic diversity as well as inbreeding and connectivity in two landscapes. We found higher levels of inbreeding and lower levels of genetic diversity in *E. myoxinus* compared with *R. rattus*. We observed related dyads both within and between habitat patches and positive spatial autocorrelation at lower distance classes for both species, with a stronger pattern of spatial autocorrelation in *R. rattus*. Across each site, we identified contrasting migration rates for each species, but these did not correspond to habitat–matrix dichotomies. The relatively low genetic diversity in the endemic *E. myoxinus* suggests ecological constraints that require further investigation.

## 1. Introduction

The island of Madagascar is a globally important biodiversity hotspot with high rates of endemism [1,2,3]. Yet, habitat loss, mainly via deforestation, poses an urgent threat to the conservation of biodiversity, in particular to endemic species on the island [4,5]. Since the 1950s, Madagascar has lost approximately 44% of its total forest area, leading to a patchwork of habitat fragments exposed to edge effects and surrounded by non-habitat matrix [6]. Generally, as habitat area decreases, community-level species richness and the abundance of individual species will decrease [7,8,9]. Following habitat loss in fragmented landscapes, a greater proportion of the remaining habitat is subject to edge effects, which can threaten populations of edge-sensitive species, a potentially confounding factor as habitat loss and increasing edge ratios occur synergistically [10,11,12]. Alternatively, edge-tolerant or ecologically flexible species may see positive outcomes following processes such as forest loss as more diverse habitat types become available [10,13]. Malagasy mammals do not respond to forest loss and fragmentation in uniform ways [14,15]. Species-specific responses to edges [16,17], vagility between remaining habitat patches [18,19], and competition with invasive species [20,21] are important research topics relevant to researchers and conservation practitioners alike in Madagascar.

When organisms are subjected to habitat loss and fragmentation, genetic and genomic methods are useful to explore and quantify negative outcomes on population genetic compositions [22,23]. Populations in fragmented landscapes may be subject to decreased gene flow between isolated subpopulations, which can lead to decreased genetic diversity across a landscape and increased genetic drift in isolated fragments [24]. These processes can lead to loss of genetic diversity through drift and inbreeding and eventually to the fixation of deleterious alleles within populations [25,26]. Increasing proportions of edge habitats in fragmented landscapes may further affect the connectivity and genetic diversity of organisms. For example, Radespiel et al. [27] found local genetic differentiation in a small-bodied lemur species, *Microcebus ravelobensis*, between edge and interior habitats within the same forest. However, the negative effects of smaller and more isolated populations may be mitigated if connectivity is high within landscapes and organisms are able to disperse across matrix and edge zones between habitat patches [28,29]. After processes such as deforestation, the new habitat types that arise in the matrix may facilitate dispersal of some organisms, in particular ecologically flexible and/or invasive species [30].

To evaluate the effects of habitat fragmentation, our study focused on two species of rodents in northwestern Madagascar: the endemic western tuft-tailed rat (*Eliurus myoxinus*) and the invasive black rat (*Rattus rattus*). As a geographically widespread species across Madagascar, *E. myoxinus* are found throughout the western and northern dry deciduous forests [31,32]. They are a small-bodied (~60 g), solitary, nocturnal mammal with a primarily arboreal lifestyle, use nests, burrows, and tree holes as sleeping sites, and consume mainly fruits [33,34,35]. Other basic natural history information such as dispersal patterns and distances are still lacking for *E. myoxinus*. As with other Malagasy fauna, studies on forest loss and fragmentation have found mixed or unclear effects. Species-specific and regional differences appear to play a large role in determining the direction and scale of effect, rather than a single unifying ecological pattern. For example, capture rates of *Eliurus* spp. increased with increasing forest area in some dry forest sites [20,33]. In other dry forest sites, no evidence for an area effect was found but signals of decreased abundance in fragmented forests were observed in relation to increasing distance from habitat edge [21]. Conversely, populations of *Eliurus* spp. in the North and West of Madagascar display relatively consistent biogeographic patterns, with weak signals of genetic differentiation from large-scale geographic barriers such as rivers, and a lack of strong isolation by distance pattern suggesting that ecological factors such as habitat preference and elevational gradients play a more important role in the biogeography of these rodents [32,36,37,38].

In comparison, *R. rattus* is a larger (~100 g), more gregarious species with a generalized diet that is found in a variety of habitats from urban and rural human settlements to primary and secondary forests [39]. When enough resources are present, *R. rattus* do not disperse over long distances, preferring to stay in close proximity to kin [40]. The introduction of *R. rattus* in Madagascar very likely occurred recently, within the last 1000 years, in port cities like Tôlanaro in the east or Antsiranana in the North via trading routes from Africa and Asia [41,42]. A complicated picture of fragmentation effects has so far been documented in *R. rattus*, perhaps due to the complicated nature of defining habitat preference for an invasive generalist species. Capture rates of *R. rattus* declined with increasing forest area in western dry forest sites [20], while forest area did not affect capture rates in other regions in the northwest and southwest [21,33]. In Central Madagascar, patterns of genetic differentiation in *R. rattus* were only weakly affected by habitat heterogeneity, and significant patterns of isolation by distance were detected as is expected in an invasive generalist [43]. However, Brouat et al. [42] found an unexpectedly weak influence of geographic distance on *R. rattus* genetic differentiation, and suggested dispersal alongside humans in a non-Euclidean pattern, such as via boats along coastlines, was responsible for the spatial genetic patterns observed. Several studies have investigated the potential for competition between introduced and endemic rodent species, but found no effect or inconclusive results [21,33]. Even if direct competition is not occurring between invasive and endemic rodents, elucidating what ecological factors allow for each of these species to persist in fragmented forest landscapes constitutes an important question from an ecological and conservation perspective.

The goal of our study was to investigate the influence of habitat loss and fragmentation on the genetic variation of these two contrasting species of rodents, *E. myoxinus* and *R. rattus*, in two fragmented forest landscapes in northwestern Madagascar. Given one arboreal, relatively specialized, and forest-dependent endemic species and one ecologically flexible, invasive species with a generalized diet that has colonized a variety of habitat types, different outcomes can be expected for these two species. We aimed to answer three main research questions for each of these two species in order to compare and contrast their movement patterns and the resulting population connectivity when living in fragmented landscapes: (1) Do endemic and invasive rodents use non-forest matrices for local movement or dispersal? (2) Are connectivity and genetic structure within these landscapes affected primarily by isolation, by distance, or by habitat-related factors? (3) Which ecological factors affect the genetic diversity of subpopulations living in fragmented landscapes?

## 2. Materials and Methods

### 2.1. Study Site and Sampling

**Figure 1:**
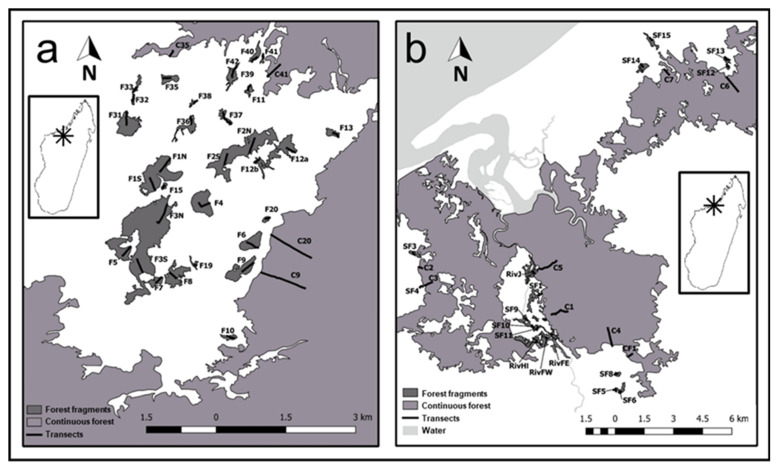
Location of study sites (**a**) ANP (Ankarafantsika National Park) and (**b**) MCF = Mariarano Classified Forest and transects (in black with name codes). Study regions were located approximately 100 km away from each other in Northwestern Madagascar (see map insets for details on location of ANP (**a**) and MCF (**b**) on Madagascar). Figure reproduced from Kiene et al. [46] (Ecology and Evolution 2021; 11:6766-6788) with permission from John Wiley & Sons Ltd., 2021.

We systematically captured animals across two landscapes in northwestern Madagascar (Mariano Classified Forest: MCF; and Ankarafantsika National Park: ANP) that contain a network of forest patches ranging in area from 0.8 to 114.6 ha, with adjacent continuous forest sites ranging from 3,683 to 130,390 ha (Figure 1, Table S1). Both landscapes are part of the dry deciduous forest ecoregion in Madagascar, an area of global conservation concern [44]. The two landscapes are separated by approximately 100 km of anthropogenically disturbed area containing settlements, rice agriculture, savanna, and grassland. Sampling occurred between June and October 2017 and 2018 during the dry season in Madagascar. While broadly similar in terms of habitat type and climate, the studied landscapes differed in several factors. The spatial scale at which we studied each landscape was different; edge-to-edge geographic distance between fragments varied from 30 m to 18,182 m in MCF and from 20 m to 6,342 m in ANP. In MCF, the forests were situated at low altitudes (10–100 m above sea level, a.s.l.) and were more humid than in ANP due to higher amounts of surface water and proximity to the ocean. In ANP (90–208 m a.s.l.), no surface water was available during the dry season, and fragments were surrounded by a relatively homogeneous matrix of grassland which has been present in its current configuration for at least 70 years [19]. In contrast, fragments in MCF were surrounded by a heterogenous savanna matrix containing varying densities of palm trees (*Bismarckia nobilis*) and a generally anthropogenic landscape featuring villages, rice fields, and roads [45]. ANP is managed by Madagascar National Parks (MNP), while MCF is managed by the Vondron’Olona Ifotony (VOI), a community-based forest protection program.

In ANP, we established 31 transects in 27 forest fragments and 4 transects in continuous forest sites. In MCF, we established 18 transects in 13 forest fragments and 8 transects across 3 patches of continuous forest (Figure 1). All transects were established perpendicular to the forest edge using a compass and a handheld GPS unit during installation. Forest edges and surface areas were defined according to Steffens and Lehman [19]. Polygons for each fragment were created by walking the perimeter of the fragment with a handheld GPS. Transects ranged in length from 40 m to 490 m into forest fragments (the shortest distance to reach the center of the fragment) and up to 1000 m in the continuous forest. We set Sherman live traps (Sherman Traps, Inc., Tallahassee, FL, USA) baited with bananas in pairs every ten meters at approximately one meter above the ground along these transects. Captured animals were anesthetized and then marked (via ear cuts), weighed, sexed, and a small biopsy was taken from the ear, placed in Queen’s Lysis Buffer, and refrigerated at 4–7 °C in the field for a maximum of six months until samples were transferred to −20 °C storage in the laboratory. For detailed description of trapping methodology and results, see Andriatsitohaina et al. [21] and Kiene et al. [47] for anesthetic procedures. We obtained an additional six samples of *E. myoxinus* collected from two other sites (Andoharano, Ambanjakely) in ANP in 2004 for comparative spatial analyses; see Rakotondravony and Radespiel [48] for further details on these study sites. We used QGIS (version 2.18.9) (QGIS Development Team, Open Source Geospatial Foundation Project, http://qgis.osgeo.org (accessed on 15 April 2022)) to determine the distance to the next edge, distance to the next continuous forest, and forest area for each individual animal captured.

All field protocols were reviewed and approved by the appropriate ethics committees at the University of Toronto, Canada (Protocol # 20011950) and the Institute of Zoology, University of Veterinary Medicine Hannover, Foundation, Germany. All fieldwork adhered to the legal requirements of Madagascar and were approved by the Ministère de l’Environnement, de l’Ecologie et des Forêts and Madagascar National Parks (Permits # 81/17/MEEF/SG/DGF/DSAP/SCB.Re, #151/17/MEEF/SG/DGF/DSAP/SCB.Re, #82/18/MEEF/SG/DGF/DSAP/SCB.Re, #92/18/MEEF/SG/DGF/DSAP/SCB.Re).

### 2.2. Laboratory Procedures and Data Preparation

We used restriction site-associated DNA sequencing (RADseq) to generate genetic data for the population genetics analyses [49]. RADseq generates sequence datasets for a large number of sites across the genome and is generally useful for the study of non-model organisms, as no prior genetic information is needed [50]. DNA was extracted from ear biopsies using a DNeasy Blood & Tissue Kit (Qiagen) following an optimized version of the manufacturer’s protocol. DNA concentrations were estimated using the Fluorometer dsDNA HS Assay Kit (Qubit) to select samples of a sufficiently high concentration. We obtained 93 samples of *E. myoxinus* (*n* = 77 individuals from ANP, *n* = 16 individuals from MCF) and 106 samples of *R. rattus* (*n* = 49 individuals from ANP, *n* = 57 individuals from MCF) for RADseq.

RADseq libraries were prepared using 100–200 ng of genomic DNA and the TruSeqNano DNA HT kit (Illumina). The restriction enzyme SbfI was used to generate DNA fragments. Libraries were prepared in sets of 24 samples sorted based on their measured DNA concentration. Each sample was ligated to one of 48 available P1 adapters with a unique five base pair molecular identifier allowing individual identification of the samples after sequencing. All fragments were randomly sheared, resulting in fragments with an average size of 550 bp. We ligated sheared DNA fragments to the P2 adapter, and all fragments with both P1 and P2 adapters were amplified in 10 polymerase chain reaction cycles. We verified DNA concentration and fragment sizes of the amplified libraries using a Qubit Fluorometer and a Bioanalyzer 2100. Finally, we sequenced the libraries using 150 bp paired-end reads on an Illumina HiSeq3000 at the GeT-PlaGe platform (Toulouse, France).

Due to a lack of a reference genome for *E. myoxinus*, we used a de novo alignment method in Stacks vs. 2.5 [51] (https://catchenlab.life.illinois.edu/stacks/) to call SNPs. We first filtered out low quality reads using *process_*r*adtags*, *clone_filter,* and *kmer_filter*. We created a catalog and aligned our samples using the de novo_*map* pipeline in Stacks 2.5 utilizing *ustacks*, *sstacks*, and *tsv2bam*. We set m, M, and *n* parameters to two, seven, and eight after optimization using protocols from Rochette and Catchen [52]. Lastly, we ran *gstacks* and *populations* retaining only loci found in at least 80% of samples (r = 0.80), with a minor allele frequency of at least 5% (min_maf = 0.05), and a maximum heterozygosity of 0.7 (max_het = 0.7), and we retained only one random single nucleotide polymorphism (SNP) per RAD locus (--write_random_snp) in order to avoid linkage disequilibrium in downstream analyses. After filtering, we retained 66 samples of *E. myoxinus*, 52 from ANP and 14 from MCF (Table S2).

We used a similar protocol and parameters for *R. rattus* whenever possible, but with a reference genome available. In Stacks 2.5 [51], we used *process_*r*adtags*, *clone_filter*, and *kmer_filter* to filter out low-quality reads. We then aligned our samples of *R. rattus* to the reference genome of congener *R. norvegicus* (Rnor_6.0, GCA_000001895.4) using the Burrows–Wheeler alignment (BWA) with BWA-MEM vs. 0.7.17 [53] (https://github.com/lh3/bwa). We then ran *gstacks* and removed read pairs of the same length (--rm-PCR-duplicates). We ran *populations,* retaining only loci found in at least 80% of samples (r = 0.80), with a minor allele frequency of at least 5% (min_maf = 0.05) and a maximum heterozygosity of 0.7 (max_het = 0.7), and we retained one random single nucleotide polymorphism (SNP) per RADtag (--write_random_snp). After filtering, we retained 81 samples of *R. rattus*, 43 from ANP and 38 from MCF (Table S2).

**Figure 2:**
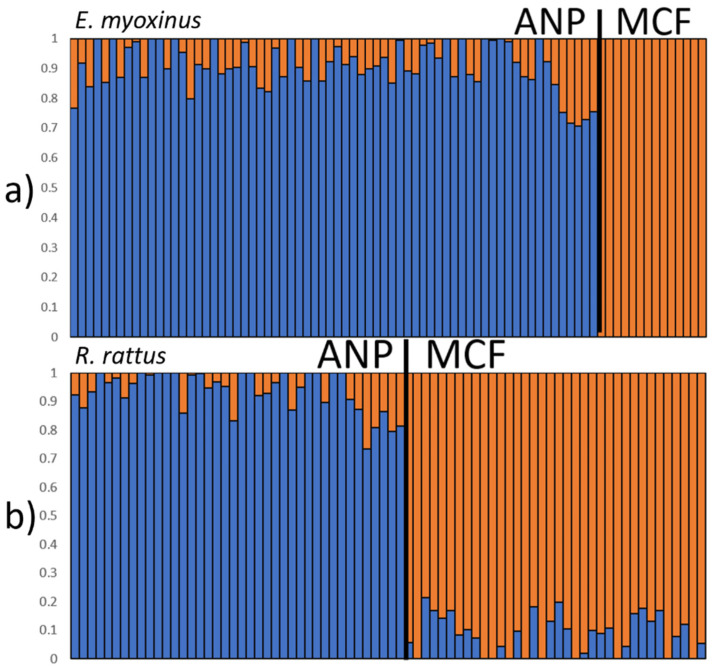
ADMIXTURE plots (results for K = 2) for *E. myoxinus* (**a**) and *R. rattus* (**b**) with both landscapes pooled to test for admixture between landscapes. Each bar in a bar plot represents one individual and its proportional assignment to the blue (ANP) and orange cluster (MCF), respectively. All individuals were analyzed together for each species but were sorted based on geographic origin in ANP (**left**) or MCF (**right**) to showcase distinction between the two sites.

As a first test to assess if each of the two landscapes contained distinct populations, we ran ADMIXTURE 1.3.0 [54] (http://dalexander.github.io/admixture/). We tested a varying number of ancestral populations (K = 2–10) and calculated cross-validation errors (CV) for each K-value to determine the most likely K. We then used the CLUMPAK program 1.1 [55] (http://clumpak.tau.ac.il/) to infer cluster assignments across 20 independent runs of ADMIXTURE 1.3.0, which revealed that each landscape contained a distinct population with comparably low levels of admixture between them (*E. myoxinus*: <30%, *R. rattus*: <27%) (Figure 2, Table S3). Therefore, all subsequent analyses were performed separately for each species in each landscape without further modifications of the SNP set. Due to limited sample sizes once species were separated at each landscape, no additional sub-analyses were completed (such as by sex or fragmented and continuous populations).

### 2.3. Analyses of Local Movement and Dispersal

We calculated a coefficient of genetic relatedness for each dyad for each landscape separately to investigate the distribution of related individuals in space. Distribution of related individuals accurately reflects the dispersal patterns within a species and can be used to detect potential dispersal events [56]. We generated pairwise relatedness values by multiplying the kinship coefficient from the *relatedness2* function in VCFtools 0.1.16 [57] (https://vcftools.github.io/) by two, and assigned dyads into discrete kinship classes based on these r values. Classes were defined as first-order relatives (r ≥ 0.375), second-order relatives (0.375 > r ≥ 0.1875), or distantly related individuals (0.1875 > r > 0) in congruence with Combs et al. [58], being aware that these classes represent approximations as we did not directly test for kinship categories but divided continuous relatedness values into three non-overlapping size classes. Dyads with r ≤ 0 were considered unrelated. We calculated geographic distances for all related dyads and calculated the mean, median, and range of geographic distances for each kinship class. We ran Mann–Whitney U tests in R Studio 1.4.1103 (R Studio Development Team, PBC, Boston, MA, USA http://www.rstudio.com/ (accessed on 10 January 2021)) to test for significant differences in geographic distances between second-order relatives and distantly related dyads within and between each species. First-order dyads were not statistically analyzed in both species due to low sample sizes.

We additionally created a genetic dissimilarity matrix using the BED2DIFFS_V1 function, which calculates average genetic dissimilarity based on allelic differences observed between samples [59] and generates a geographic distance matrix (Ersts, American Museum of Natural History, Center for Biodiversity and Conservation, http://biodiversityinformatics.amnh.org/open_source/gdmg (accessed on 19 April 2021)). We used these two matrices to create Mantel correlograms using the *mantel.correlog* function in the Vegan R package 2.6-4 with 999 permutations [60]. Mantel correlograms test for spatial autocorrelation between genetic and geographic distances in a predefined set of distance classes. We selected distance classes of 200 m width due to the expectation for short dispersal distances in *R. rattus* [61], and used the same distance classes for *E. myoxinus* for comparability. Only dyads from individuals of less than half the maximum Euclidean geographic distance were included in this analysis to reduce a potential bias due to individuals from the edges of the sampling space [62].

### 2.4. Analyses of Connectivity and Genetic Structure on the Landscape Level

In order to assess the effect of isolation by distance (IBD) across the entire population in each landscape, we first conducted Mantel tests using the genetic dissimilarity matrix and the geographic distance matrix described above. We ran Mantel tests with 999 permutations on each species and landscape separately using the Vegan R package 2.6-4 [60].

We used three complementary methods to determine sub-population connectivity and genetic structure within the two landscapes. For all three analyses, we used a slightly reduced dataset with one individual of each closely related dyad being removed (*n* = 4 individuals removed). First, we ran ADMIXTURE 1.3.0 [54] to assess genetic structure via ancestry estimation across each landscape using the same methods described above on each species and landscape independently. We pooled population ancestry estimates from ADMIXTURE for all individuals from each forest site into one pie chart to visualize population structure across the landscape.

Secondly, we used the Estimating Effective Migration Surfaces (EEMS) software package 0.0.0.9000 (https://github.com/dipetkov/eems) to investigate migration rates and potential barriers to gene flow across each landscape. EEMS uses a stepping-stone model to estimate effective migration rates between adjacent subpopulations (demes) and produces a visual migration surface of estimated genetic barriers and connectivity [59]. Specifically, the program highlights geographic areas where genetic distance between samples increases more quickly (i.e., low genetic connectivity) or more slowly than expected (i.e., high genetic connectivity). Following Barratt et al. [63], we used the genetic dissimilarity matrix and raw geographic coordinates for each sample as input files for EEMS. We ran EEMS using deme sizes of 1000 (the spatial resolution of the analysis) after evaluating the effect of varying resolution (250, 500, and 750, results not shown). Running EEMS with various deme sizes showed similar relationships across the landscapes and species, but due to their highest spatial resolution, we only present results from the runs with 1000 demes. We produced migration surfaces for each species on each landscape separately and produced additional migration surfaces for *R. rattus* for the Northern and Southern patches of forest in MCF due to the large spatial scale of the site and sufficient sample size. Our analyses used two MCMC chains, each with a length of 25,000,000 iterations, with a burn-in of 1,000,000 iterations. We examined the log posterior between the chains and combined results from both using the rEEMSplot package 0.1.0. We then plotted surfaces of effective migration rates across each landscape to identify geographic barriers and areas of connectivity.

Thirdly, we ran the ResDisMapper program 1.1 [64] (https://rdrr.io/github/takfung/ResDisMapper/man/ResDisMapper-package.html), which estimates resistance to dispersal across landscapes. ResDisMapper 1.1 calculates IBD residuals for each dyad of individuals and maps resistance values based on their deviations from the general IBD trend. Genetic data were input into ResDisMapper 1.1 using the GENEPOP format, and a Euclidean distance matrix was used to produce IBD residuals using the “relative similarity method” (Method 1). We ran ResDisMapper 1.1 with default values of a final resolution of 50, confidence intervals set to 0.95, for 1,000 repetitions. The significance threshold for resistance contours was set to 0.05. For MCF, we set the maximum distance for dyads at 10,000 m to ensure a continuous distribution of IBD values [64], as sampling did not occur continuously across MCF. Our samples from ANP only included dyads less than 5,000 m apart sampled continuously across the landscape. We used the *rdm_mapper* function to produce a resistance map which was then overlaid on a map of each landscape produced in QGIS 2.18.9.

### 2.5. Ecological Determinants of Genetic Diversity

We calculated three parameters of genetic diversity to determine which ecological factors affected genetic diversity of the two rodents across the landscapes. The three individual-based parameters were the inbreeding coefficient (F_IS_), observed heterozygosity (H_O_), and expected heterozygosity (H_E_). These three parameters were calculated using the VCFtools --*het* command [57] for each individual *E. myoxinus* and *R. rattus*. Generalized additive models (GAMs) were built for each of these dependent variables (Link function = identity, family = Gaussian) using maximum likelihood estimation in the R package mgcv 1.8.42 [65] and the fixed factors species (*E. myoxinus*, *R. rattus*), region (ANP, MCF), sex (male, female), forest type (continuous, forest fragment), body mass (a proxy for body condition), forest area (of the fragment where an individual was captured), distance to edge (of an individual to the nearest forest edge), distance to continuous forest (of a fragment edge to the nearest continuous forest edge, a proxy for isolation), and the percentage of edge habitat (area of the fragment within 50 m of the forest edge). Normality of all continuous variables was assessed using Shapiro–Wilk’s tests [66]. If a variable was not normally distributed, it was transformed using either log or square root transformations, and Q-Q plots were visually inspected to ensure improvement. We tested for correlations between all continuous predictor variables (body mass, forest area, distance to edge, edge percentage, and distance to continuous forest) to ensure independence of the fixed factors included in a given model (Table S4). We modeled each genetic diversity parameter (a) with the complete dataset (*n* = 123 individuals) and (b) just with the individuals caught in forest fragments (*n* = 90 individuals) to also test for variables that are not relevant for continuous forest sites (i.e., ‘percentage edge habitat’ and ‘distance to continuous forest’). The separation of correlated variables into different models resulted in two GAMs built for the overall data set (Models A-B) and three GAMs built for the fragment-only data set (Models C–E, Table 1). Additionally, we ran each model for each species separately to control for the potentially confounding interaction of species with other variables.

We determined the best model for each full model A–E using a model-averaging algorithm implemented in the “dredge” command from the R package MuMIn 1.47.5 [67]. This process explores all possible combinations of factors in a given model and ranks them according to Akaike’s Information Criterion value [68]. We considered as best models those with a low corrected Akaike’s Information Criterion (ΔAICc < 2) and the highest AICc weight (ω) [68]. We used local regression smoothing and Loess smoothing to visualize nonlinearities in the models [69].

## 3. Results

### 3.1. RADseq Dataset

After filtering with the STACKS pipeline, we recovered 42,855–59,901 RAD loci per individual with a mean depth of 9.79x coverage (5.05x–23.82x per individual) for *E. myoxinus* individuals (*n* = 52) from ANP, and 53,070–69,657 RAD loci per individual with a mean depth of 10.09x coverage (8.57x–12.39x per individual) for *E. myoxinus* individuals (*n* = 14) from MCF (Table S2). We recovered 40,542–49,097 RAD loci per individual with a mean depth of 9.68x coverage (5.40x–19.43x per individual) for *R. rattus* individuals (*n* = 43) from ANP, and 34,451–48,855 RAD loci per individual with a mean depth of 10.42x coverage (5.90x–15.04x per individual) for *R. rattus* individuals (*n* = 38) from MCF. SNP sets were generated for each species separately and individuals were grouped for different downstream analyses based on need (Table S2).

### 3.2. Patterns of Local Movement and Dispersal

**Figure 3:**
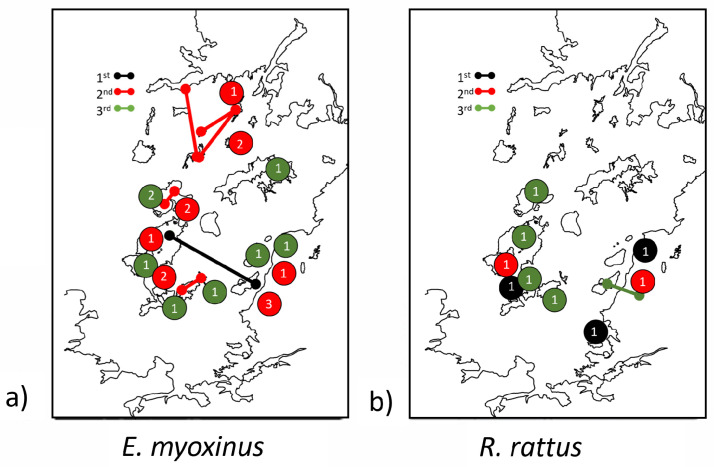
Geographic distribution of all related dyads (r > 0) at ANP (Ankarafantsika National Park) for (**a**) *E. myoxinus* and (**b**) *R. rattus*. Related dyads are sorted into first-order relatives (r ≥ 0.375, black), second-order relatives (0.375 > r ≥ 0.1875, red), and third-order relatives (0.1875 > r > 0, green). Lines illustrate the Euclidian distance between two related individuals that were captured on separate transects, mostly separated by matrix, while closed circles represent the location and number of related dyads in which both individuals were captured along the same transect.

We identified 26 related *E. myoxinus* dyads at ANP (r > 0.0, 1.5% of all possible dyads, Table 2), including two first-order relatives (r ≥ 0.375) that were captured 2.17 km apart in different forest patches (the two patches were separated by ~1.5 km of matrix), suggesting a relatively recent long-distance dispersal event across the grassland savanna matrix (Figure 3). All third-order relatives were found further apart from each other (494 m–2.5 km apart) than second-order relatives (up to 294 m). While the partners from 12% (2/17) of the second-order dyads were found in different forest patches, this was the case for 88% (7/8) of the distantly related dyads (Figure 3). No related (r > 0.0) *E. myoxinus* individuals were identified at MCF, but sample size was rather limited (*n* = 14).

We identified 10 related dyads in *R. rattus* at ANP (r > 0.0, 1.1% of all possible dyads) and 6 at MCF (r > 0.0, 0.85% of all possible dyads, Table 2). All first- and second-order relatives of *R. rattus* (r ≥ 0.1875) were trapped within 110 m of each other, while distantly related *R. rattus* (r < 0.1875) were trapped within 760 m of each other. Related *R. rattus* dyads were trapped in different forest patches in only one case (10%, 1/10, Figure 3).

In both *E. myoxinus* and *R. rattus*, second-order relatives were captured in closer proximity to each other than distantly related dyads (*E. myoxinus*: U = 16, *p* < 0.001 *R. rattus*: U = 28, *p* = 0.006). For both second-order and distant relatives, *R. rattus* dyads were in closer proximity to each other than the respective *E. myoxinus* dyads (second-order relatives: U = 136, *p* < 0.001, distant relatives: U = 26, *p* < 0.001).

**Figure 4:**
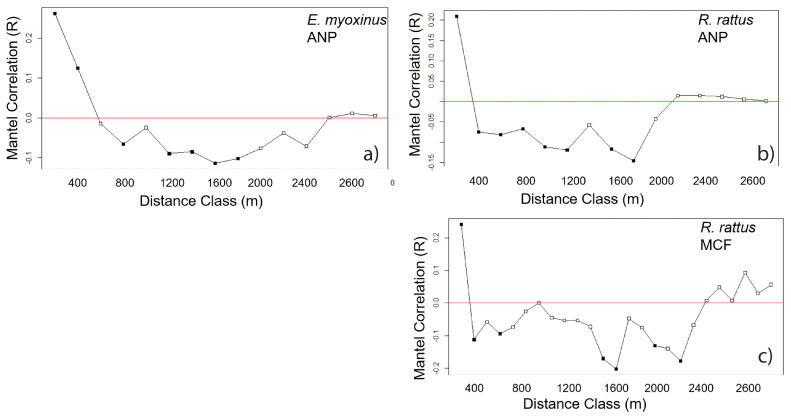
Mantel correlograms for *E. myoxinus* (**a**) and *R. rattus* (**b**,**c**) in each landscape, with pairwise dyads sorted into distance classes of 200 m width on the *x*-axis and the Mantel Correlation Coefficients (R) on the *y*-axis. Filled squares indicate significant correlations (*p* < 0.05), open squares indicate non-significant correlations (*p* > 0.05), and the red line is positioned at a Mantel Correlation Coefficient of R = 0. Results for *E. myoxinus* from MCF are not shown as no significant values were found due to small sample size. ANP = Ankarafantsika National Park, MCF = Mariarano Classified Forest.

We found significant effects of spatial autocorrelation with Mantel correlograms in *E. myoxinus* at ANP and for *R. rattus* at both ANP and MCF, but spatial autocorrelation could not be tested for *E. myoxinus* at MCF due to small sample size (Figure 4). For *E. myoxinus* at ANP, spatial autocorrelation between genetic distance and geographic distance was significantly positive for the first two distance classes (0–200 m, 200–400 m, Figure 4), while for *R. rattus* at ANP and at MCF, spatial autocorrelation was significantly positive only at the first distance class (0–200 m) (Figure 4).

### 3.3. Connectivity and Genetic Structure on the Landscape Level

**Figure 5:**
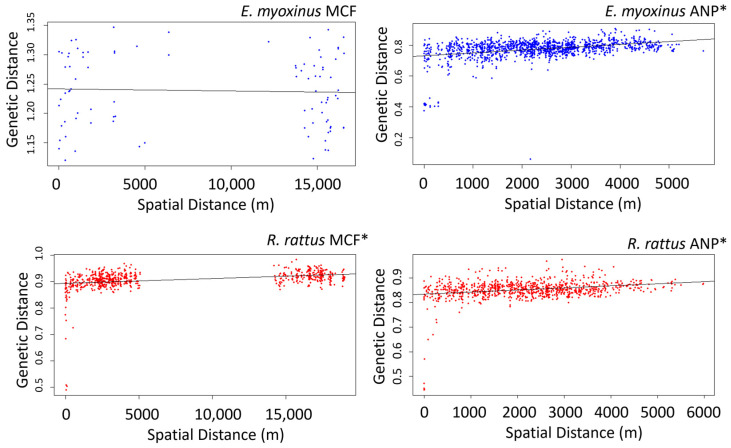
Relationship between geographic and genetic distance for all individuals of *E. myoxinus* (**a**,**b**) and *R. rattus* (**c**,**d**) in each landscape. The *x*-axis displays the Euclidean spatial distance between individuals (in meters) and the *y*-axis displays the genetic distance between individuals. ANP = Ankarafantsika National Park, MCF = Mariarano Classified Forest. * indicates significant correlation revealed by Mantel tests (*p* < 0.05).

Significant IBD effects were identified in both species (Figure 5). Genetic distance increased with increasing geographic distance in *E. myoxinus* at ANP (Mantel test, r = 0.222, *p* < 0.01, *n* = 52), and in *R. rattus* at both ANP (Mantel test, r = 0.4399, *p* < 0.01, *n* = 43) and MCF (Mantel test, r = 0.3211, *p* < 0.01, *n* = 37). No such effect was detected for *E. myoxinus* at MCF (Mantel test, r = −0.02, *p* = 0.52, *n* = 14), possibly due to small sample size.

**Figure 6:**
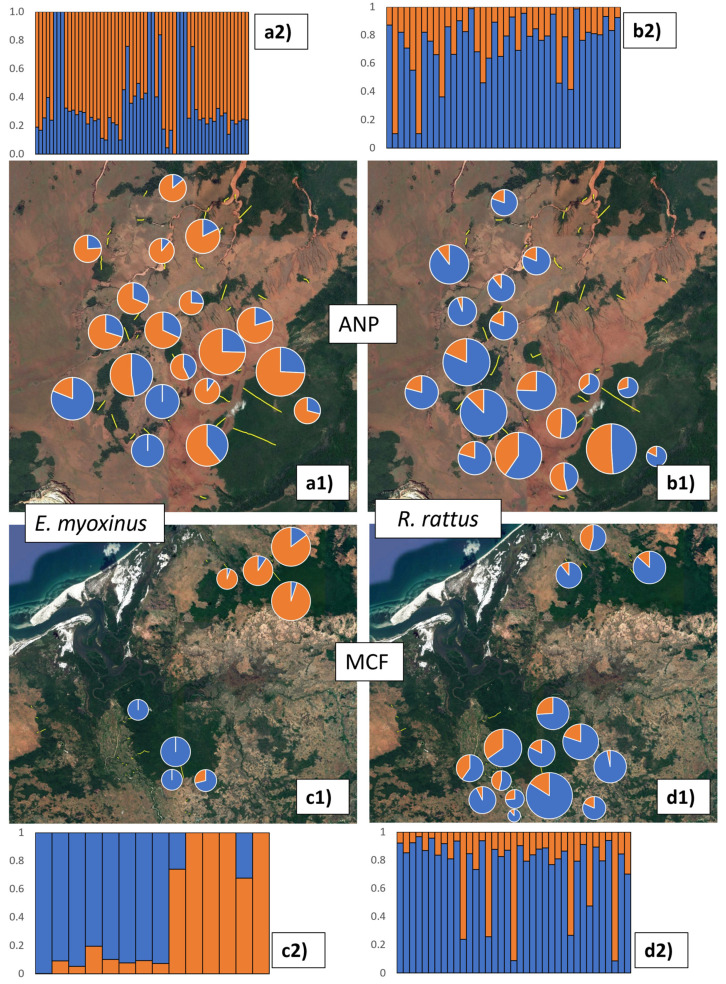
Relative representation of two ADMIXTURE clusters (K = 2, blue and orange) that were determined separately for each species and landscape. Pie charts were generated for the different study sites within each landscape for *E. myoxinus* (**a1**,**c1**) and *R. rattus* (**b1**,**d1**) based on the sum of all individual, proportional admixture values for each site. The relative size of each pie chart illustrates the number of individuals sampled within each site. The underlying individual assignment proportions to the blue and orange cluster are displayed in barplots above (ANP; **a2**: *E. myoxinus*,**b2**: *R. rattus*) or below (MCF; **c2**: *E. myoxinus*,**d2**: *R. rattus*) the respective map. Each bar in a bar plot represents one individual and its proportional assignment to the blue and orange cluster, respectively. ANP = Ankarafantsika National Park, MCF = Mariarano Classified Forest.

Separate ADMIXTURE analyses for each landscape and species suggested an optimum of one or two clusters for each species within each landscape (see Figure S1 for justification and for results K = 3), of which we plotted the relative representation of K = 2 clusters in space (Figure 6). There was no strong signal of genetic structure in *E. myoxinus* in ANP, although one cluster (= blue) was dominant in the southwestern part of the landscape and rarer in the other parts (Figure 6a1). At MCF, the southern and northern *E. myoxinus* subpopulations fell into largely separate clusters, with individuals showing only low proportions of admixture (Figure 6c1). In contrast, there was no clear signal of genetic structure in *R. rattus* in either study region (Figure 6b1,d1).

**Figure 7:**
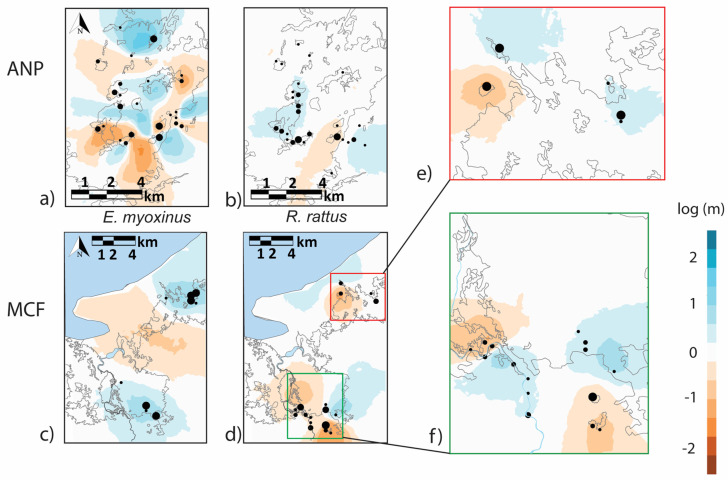
Estimated migration surfaces generated with EEMS for *E. myoxinus* (**a**,**c**) and *R. rattus* (**b**,**d**) on each landscape with additional sub-analyses performed for *R. rattus* from the southern and northern sections of MCF separately (**e**,**f**). Blue shading represents high effective migration rates (inferred migration corridors), while orange shading represents low migration rates (inferred migration barriers). The relative size of each point illustrates the number of individuals sampled in each location.

At ANP, effective migration surfaces for *E. myoxinus* suggested highly variable connectivity across the landscape (Figure 7a). High effective migration rates (log(m) ≥ 0, = blue parts) were inferred within the central and northern sections of the fragmented landscape and in parts of the eastern continuous forest. In contrast, lower effective migration rates (log(m) < 0, = orange parts) were inferred for an area encircling the central part of high connectivity and for one stretch of the eastern continuous forest. At MCF, the few available samples came mainly from the two continuous forest sites in the North and in the South. We inferred high effective migration rates of *E. myoxinus* within each continuous forest site, while the savanna between the two forests acted as a barrier to gene flow (Figure 7c). At ANP, for *R. rattus*, a reasonably high effective migration rate was inferred for large parts of the fragmented landscape and in the eastern continuous forest (Figure 7b). A low effective migration rate was only inferred between these two areas. At MCF, the effective migration surfaces showed a more complex picture for the northern and southern sites, respectively (Figure 7d). In the northern sub-section (Figure 7e), only a small number of individuals were present (*n* = 8) with mixed results; between two fragments, there was a low effective migration rate, while between another set of fragments and the continuous forest, there were high effective migration rates. In the southern forest sites (Figure 7f), there was a high effective migration rate amongst individuals in the continuous forest and along a section of the Mariarano river, but low rates were inferred amongst individuals in the forest fragments of the south and the west.

**Figure 8:**
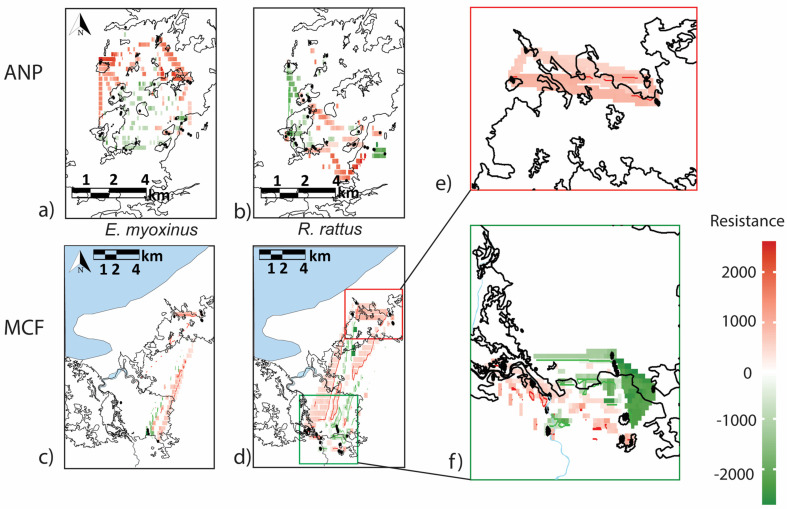
Landscape resistances values generated with ResDisMapper 1.1 for *E. myoxinus* (**a**,**c**) and *R. rattus* (**b**,**d**) on each landscape with additional sub-analyses performed on *R. rattus* from the southern and northern sections of MCF separately (**e**,**f**). Red shadings signify high resistance to movement (=low gene flow), while green shadings signify low resistance values (=high gene flow). Significantly positive or negative resistance values are indicated by dark red or green lines.

ResDisMapper 1.1 also detected heterogeneous patterns of landscape resistance across the two study regions for the two species (Figure 8), with positive resistance values (green) indicating high levels of connectivity and negative resistance values (red) indicating low connectivity [64]. At ANP, areas of significantly high and low landscape resistance were inferred for *E. myoxinus* (Figure 8a). Overall, the Central and Southern fragments were all characterized by very low resistance, while very high resistance values were inferred towards and between the Northern fragments. At MCF, there were no significant resistance levels detected for *E. myoxinus*, but the individuals in the southern continuous forest had low resistance values, while the individuals in the north had higher values (Figure 8c). At ANP, for *R. rattus*, we detected significantly low resistance values in the western fragments and the eastern continuous forest, while significantly high resistance values were detected in the southern and central fragments (Figure 8b). At MCF, we detected significantly low resistance values for individuals in the southern continuous forest and along the Mariarano river, while we detected significantly high resistance values amongst the western fragments and in and among the northern forest patches (Figure 8d). In the northern sub-section (Figure 8e), there were only positive resistance values for the individuals within and between forest patches. In contrast, at the southern forest sites (Figure 8f), there were negative resistance values amongst individuals in the continuous forest and along one section of the Mariarano river, but also positive resistance values between fragmented forest sites and along other sections of the river.

### 3.4. Ecological Determinants of Genetic Diversity

**Figure 9:**
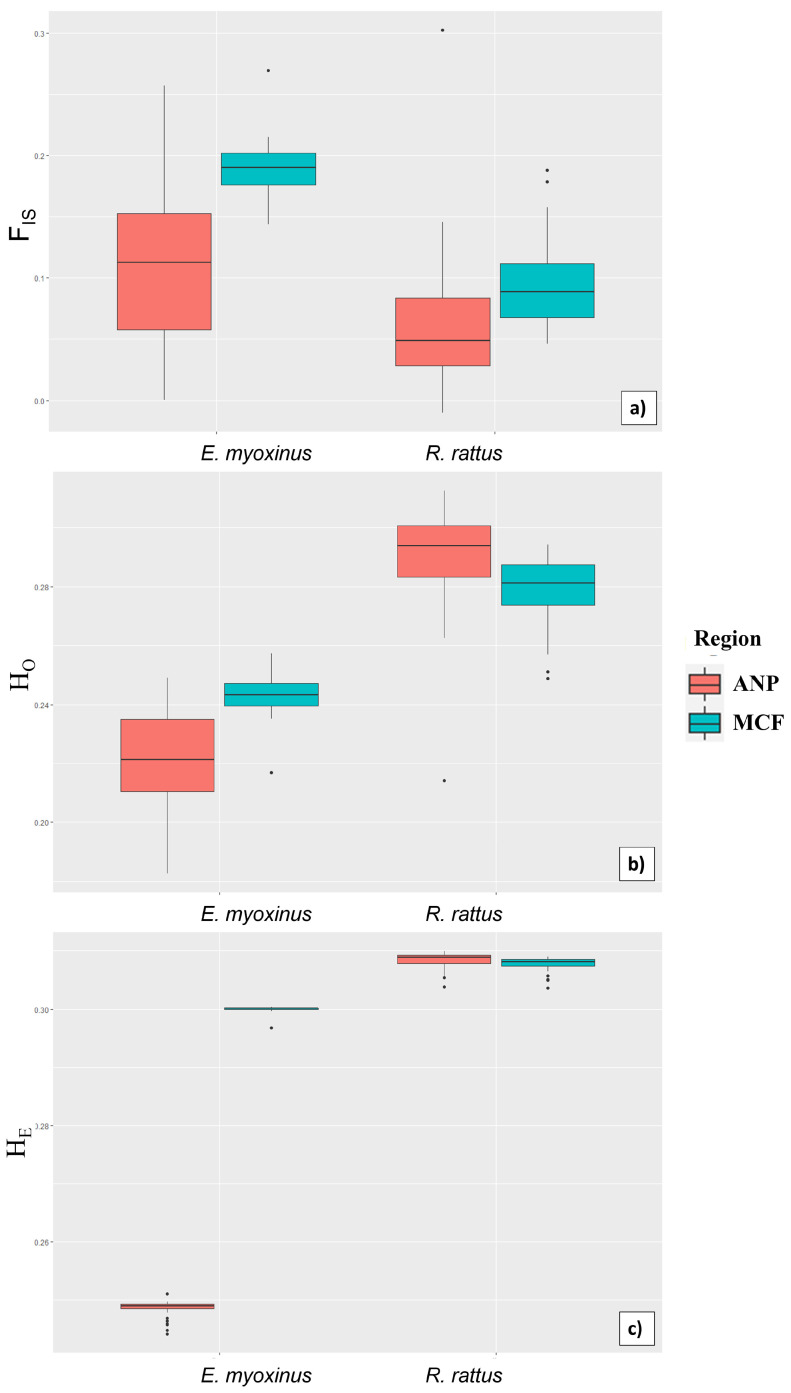
Comparison of the inbreeding coefficient (F_IS_, (**a**)), observed (H_O_, (**b**)) and expected heterozygosity (H_E_, (**c**)) on the *y*-axis between *E. myoxinus* (**left**) and *R. rattus* (**right**) in both regions (orange = ANP, green = MCF). Medians, quartiles (boxes), minimum/maximum without outliers (whiskers), and outliers (black dots) are displayed. ANP = Ankarafantsika National Park, MCF = Mariarano Classified Forest.

Across all models and among all variables, region and species had the strongest impact on genetic diversity (Table 3 and Table S6). Overall, *E. myoxinus* had significantly higher inbreeding coefficients (F_IS_) and significantly lower observed and expected heterozygosity than *R. rattus* (Figure 9, Table 3). The inbreeding coefficient F_IS_ was significantly higher at MCF than in ANP in both species (Figure 9, Table 3 and Table 4). However, while observed and expected heterozygosity in *E. myoxinus* were higher at MCF than at ANP, the opposite regional effect was detected in *R. rattus*, with higher values at ANP for both measures of heterozygosity (Figure 9, Table 4). Among all ecological variables, only the distance to edge had significant effects on genetic diversity. First, it was positively associated with the inbreeding coefficient in the overall sample (Table 3). Second, observed heterozygosity decreased significantly with increasing distance to edge in *E. myoxinus*, but not in *R. rattus*. No other parameter had any significant influence on genetic diversity in any model (Table 3 and Table 4).

## 4. Discussion

### 4.1. Do Endemic and Invasive Rodents Use Non-Forest Matrices for Local Movement or Dispersal?

The spatial distribution of related individuals suggests that endemic *E. myoxinus* are able to move between isolated patches of forest across the matrix, despite their primarily arboreal lifestyle [33]. Several relatives (including one first-order dyad that may suggest a parent–offspring or full sibling relationship) were separated by a savanna matrix at ANP, which implies dispersal occurred across the matrix. The ability of *E. myoxinus* to cross the matrix may also explain why this and previous studies have found individuals in small fragments that have been surrounded by the matrix for at least 70 years and thereby at least 70 generations of *E. myoxinus* [21], although the ability to disperse to these isolated fragments does not necessarily ensure long-term persistence. The Mantel correlograms for *E. myoxinus* showed significant positive correlations at low distance classes, followed by negative correlations at higher classes, suggesting some degree of local clustering of related individuals. However, both the steep drop-off of correlations after 400 m and the negative correlation values beyond suggest dispersal capacity is reduced in a fragmented landscape. When habitat is continuous and dispersal is unconstrained, spatial correlation would be expected to decline in a more gradual pattern [70], and negative correlation values have been linked with reduced dispersal due to habitat disturbance [71]. Although the dispersal distances of *E. myoxinus* in continuous forest habitats are not yet known, our results suggest that despite reduced dispersal ability in fragmented landscapes, *E. myoxinus* maintains some connectivity between adjacent forest fragments and via opportunistic long-distance dispersal events across the matrix.

The invasive *R. rattus* showed different patterns of relatedness and dispersal across the landscapes. Related dyads were found in neighboring forest patches only in a few cases (2/16 cases), we did not observe any long-distance dispersal events, and all closely related dyads (r ≥ 0.1875) were captured within ~100 m of each other. These results suggest philopatric tendencies and kin-biased spatial clustering in *R. rattus* which has already been described from other study sites in Madagascar [40]. This finding is also supported by the results of the Mantel correlograms as significantly positive spatial autocorrelation could only be shown at short distances (<200 m). In a study on congener *R. norvegicus* in an urban landscape, a decline in correlations was also observed beyond 200 m but in a much more gradual pattern [58]. Correlograms with steep declines and significant negative values may again be interpreted as filtering effects of fragmentation as in the case of *E. myoxinus* [70,71]. Thus, dispersal of *R. rattus* in our study seemed to be reduced due to fragmentation as well, although dispersal across the matrix was possible but probably relatively rarer due to a preference for staying close to their natal site and not necessarily a lesser ability to cross matrix than in *E. myoxinus*. While *R. rattus* are generally able to exploit a breadth of habitat types, our results indicate that they do not use savanna as habitat, although it is clearly permeable to dispersal.

We found related *R. rattus* dyads in closer proximity than related *E. myoxinus* dyads, which was unexpected, as invasive species tend to have greater dispersal abilities than native species [42]. These differences may be attributed to differences in social organization and resource requirements between the two species, rather than in dispersal ability per se. *E. myoxinus* is a solitary, frugivorous, arboreal rodent [33,34] and was found in lower numbers in this fragmented landscape than *R. rattus* [21]. In contrast, *R. rattus* forms gregarious groups of related members and has an opportunistic, omnivorous diet [40]. While the longer dispersal distances of *E. myoxinus* are in concordance with the solitary lifestyle of a dietary specialist [33], their lower reported abundance in fragments may rather be attributed to specific resource and microhabitat requirements than to a general lack of vagility between the continuous forest and/or forest fragments. In contrast, *R. rattus* seems to be largely philopatric, with less dispersal across the matrix as a by-product of individuals preferring to stay near kin rather than an inability to disperse across open spaces. Thus, it is possible that the forest fragments we studied contain enough resources for *R. rattus*, while *E. myoxinus* are dispersing greater distances due to a lack of suitable habitat in fragmented landscapes. While our study did not directly test for this, it is also possible that human-mediated dispersal is occurring in these landscapes. While *R. rattus* and *E. myoxinus* both have different life history strategies, both species are able to maintain dispersal across non-habitat matrices and inhabit isolated forest fragments.

### 4.2. Are Connectivity and Genetic Structure Affected Primarily by Isolation by Distance or by Habitat?

*E. myoxinus* at ANP showed a pattern of isolation by distance. Previous research on *Eliurus* spp. did not find significant effects of IBD using mitochondrial DNA [36,37], but we examined IBD at a smaller spatial scale (5 km vs. 42–83 km) that is potentially affected by the small-scale dispersal patterns of individuals. The effect of scale can also be seen between the two landscapes, as the two *E. myoxinus* sub-populations at MCF showed strong signals of genetic differentiation between the two large forest patches separated by savanna, while a more complex patchwork of connectivity was observed at ANP, not directly connected to a matrix–forest dichotomy. This effect is in line with range-wide patterns of *E. myoxinus,* which has a wide distribution and is mainly structured by altitudinal gradients [32] with high rates of gene flow within the core of their range [34]. However, there has so far been little research done on this species at the scale of our study. The complex spatial patterns in connectivity at ANP could be due to other factors such as spatial variation in predation risk [72] or microhabitat preferences in *E. myoxinus*, which may also explain the lack of unifying ecological patterns seen in other studies [16,21,73].

*R. rattus* sub-populations at both ANP and MCF showed significant effects of IBD. However, we also observed different patterns of connectivity across the landscape, suggesting that other habitat factors such as matrix composition and quality may also structure these sub-populations. We observed two distinctive areas of high connectivity in larger forest patches at ANP and a more complicated pattern at MCF, but similar areas of high connectivity in larger forest patches. These movement patterns suggest a general preference for larger patches of forest but also may be explained by the population dynamics of *R. rattus* in the landscapes. More specifically, the high degree of genetic connectivity among the larger fragments in ANP points towards a larger and well-established population nucleus consisting of multiple, largely philopatric matrilines that are rather isolated from neighboring lineages inhabiting the smaller, isolated fragments due to a scarcity of long-distance dispersal. Anthropogenic influences may also be structuring *R. rattus* populations in the region. *R. rattus* have been observed to follow human paths and settlements rather than dispersing in a Euclidean manner [42]. There are no long-term human settlements between our capture locations at ANP, although some villages are present north and northeast of the landscape [19], while the landscape at MCF is interspersed with several villages and agricultural land [45].

From a conservation management standpoint, our study showcases the difficulty in managing invasive *R. rattus* populations in Madagascar. Eradication is unfeasible, especially in a country with limited conservation resources. *R. rattus* are able to exploit and colonize continuous forest and forest fragments likewise, and the only habitat type they seem not to use, savanna matrix, cannot be expanded as it would also negatively affect endemic species such as *E. myoxinus*. It is likely that some of habitat-related factors structuring these two species are similar, such as vulnerability to predation [72], while microhabitat requirements may vary due to differing habitat preferences and feeding habits. While we found that IBD is an important factor structuring these populations, these other habitat-related factors that are structuring populations should be examined further.

### 4.3. What Ecological Factors Affect the Genetic Diversity of Rodent Sub-Populations?

Only three ecological factors influenced the genetic diversity of the studied rodents, and these were region, species and, in the case of *E. myoxinus*, distance to edge. The regional differences in the case of *E. myoxinus* were rather unexpected, since both the inbreeding coefficient and the observed and expected heterozygosity were higher in MCF compared to ANP. These rather inconsistent findings may have been driven by the small sample size from MCF, which precludes further interpretation.

In congruence with the overall significant and positive relationship between F_IS_ and distance to edge, H_O_ decreased significantly with increasing distance to edge, but only in *E. myoxinus*. Taken together, these findings suggest that genetic diversity, at least in *E. myoxinus*, was higher closer to edges than towards the center of forests. Previous research from the same two landscapes found a decreased abundance of *E. myoxinus* in fragments with a higher percentage of edge zone, and it was concluded that *E. myoxinus* shows negative effects of habitat edges and of extensive habitat fragmentation [21]. If that is the case, a decrease of genetic diversity with increasing distance to edges may not be explained with a higher abundance of *E. myoxinus* near edges. Alternatively, the higher levels of genetic diversity near edges may have resulted from an increased diversity of mating opportunities with unrelated conspecifics near edges, since immigrants from other fragments arrive first in the edge zone of any given site, and long-distance dispersal was confirmed for *E. myoxinus* in the present study. Radespiel et al. [27] found decreased gene flow between mouse lemurs from edge and interior zones, suggesting a “local preference model” in which the ecological differences between edge and interior habitat coincide with a preference for the natal habitat. This may also be the case in *E. myoxinus* if individuals born in edge zones of fragments would rather disperse across the matrix to other edge zones than towards interior sub-populations. Such a behavior would lead to an increasing genetic diversity in edge zones, despite a lack of high-quality habitat creating an ecological trap. Molecular edge effects are understudied and represent an important upcoming topic in fragmentation research, especially given the adoption of next-generation sequencing which may be able to parse out smaller-scale patterns in genetic differentiation [74]. Given that nearly half of the remaining forest in Madagascar is within 100 m of the edge [6], major conservation concerns would arise if forest edges were confirmed to act as an ecological trap for *E. myoxinus* or other endemic rodents.

The only significant factor affecting the genetic diversity of *R. rattus* when analyzed individually was region; we observed higher levels of genetic diversity at ANP. This may reflect the invasion dynamics of *R. rattus* in northwestern Madagascar. At least two founder events occurred in Madagascar, one in the North and one in the South, followed by a relatively slow range expansion across the island. The populations in northwestern Madagascar were suggested to have resulted from the southern invasion [42,75]. Consequently, the MCF population of *R. rattus* would lie at the edge of the range of the southern expansion and thus may have been more recently colonized than ANP and undergone a founder effect, which is known to coincide with a population bottleneck and lower genetic diversity. The lack of other significant ecological impacts on genetic diversity may also suggest ecological flexibility, lack of habitat preference, and their principal ability to cross the matrix and mitigate its isolation effects. Future research, using methods such as approximate Bayesian computation (ABC) to infer demographic history of *R. rattus* could help tracing and reconstructing the original invasion(s) into Madagascar and its spread throughout the North into our study landscapes.

Genetic diversity of *R. rattus* was higher compared to *E. myoxinus* at both sites. Invasive species with a limited number of invasion points in recent history should have lower levels of genetic diversity compared with established populations of native species in a given landscape due to founder effects [76]. While previous research suggests *R. rattus* and *E. myoxinus* population abundances are not correlated in this site, suggesting a lack of competition [20,21], our results suggest that *R. rattus* are better equipped to expand into and establish viable populations within fragmented landscapes compared with *E. myoxinus*. Compared with *R. rattus*, *E. myoxinus* has more specialized habitat requirements, such as tree holes for sleeping sites and a frugivorous diet [35]. Thus, while direct competition is not necessarily occurring, the endemic *E. myoxinus* may still be at greater risk of extirpation in these fragmented landscapes over time, especially as deforestation continues and the amount of remaining habitat subject to edge effects increases. Given that distance to edges was the only significant ecological variable we found affecting *E. myoxinus*, we encourage further research in the microhabitat preference and natural history of this species in order to more effectively inform conservation.

## 5. Conclusions

We found variation in local movement, connectivity, and genetic diversity for rodents in Malagasy fragmented dry forests. Certain aspects of these species’ life histories seem to allow them to colonize and/or persist in fragmented landscapes. *E. myoxinus*, a solitary species with more specialized habitat requirements dispersed large distances between habitat patches across an open savanna matrix. However, decreased abundance, lower levels of genetic diversity overall, and lower genetic diversity with increasing distances from habitat edges suggest that the long-term persistence of this endemic species in fragmented landscapes is not secured. These overall threats are increased by the rapid rates of deforestation in Madagascar [6] and probably also affect other endangered local microendemic rodents across Madagascar, for whom there is an even greater dearth of data. Conversely, *R. rattus*, a gregarious and ecologically flexible, invasive species, is well adapted to living in fragmented landscapes of northwestern Madagascar, although it maintains connectivity with short-distance rather than long-distance dispersal. Our results add further complexity to the literature on mammal populations in fragmented forests of Madagascar and suggest further research is needed. In particular, basic natural history information (e.g., dispersal patterns and distances, sleeping habits, social organization, food resources, reproduction, microhabitat preferences) is missing for many species, which would ultimately help to improve conservation initiatives.

## Figures and Tables

**Table 1:** Summary of models used to determine the variables affecting genetic diversity. Data sets contained either all individuals (*n* = 123) or the subset of individuals captured in fragments (*n* = 90). Variables were not included in the same model if they were correlated with each other (Table S4).

Model	Data Set	Categorical Variables	Continuous Variables
A	All individuals	Species, Region, Sex	Body mass, Forest Area
B	All individuals	Species, Region, Sex, Forest Type	Body mass, Distance to Edge
C	Only individuals from fragments	Species, Region, Sex	Body mass, Forest Area
D	Only individuals from fragments	Species, Region, Sex	Body mass, Distance to Edge
E	Only individuals from fragments	Species, Region, Sex	Body mass, Distance to Continuous Forest, Percentage of Edge

**Table 2:** Summary statistics for three classes of related dyads from each species in each landscape. Number of dyads, median, and range of Euclidean pairwise distances (in meters) for first-order relatives (FOR, r ≥ 0.375), second-order relatives (SOR, 0.375 > r ≥ 0.1875), and distant relatives (DR, 0.1875 > r > 0). N: sample size, ANP = Ankarafantsika National Park, MCF = Mariarano Classified Forest. NA = not available. For further information on related dyads see Table S5.

Species	Region	N FOR	Distance (m)	Range (m)	N SOR	Distance (m)	Range (m)	N DR	Distance (m)	Range (m)
*E. myoxinus*	ANP	1	2174	2174	17	35	0–294	8	770	494–2523
	MCF	0	NA	NA	0	NA	NA	0	NA	NA
*R. rattus*	ANP	3	6	0–10	2	5	0–11	5	263	93–758
	MCF	0	NA	NA	3	62	49–110	3	75	40–520

**Table 3:** Ecological determinants of genetic diversity—results from GAMs with both rodent species modelled together. Black shading indicates variable was not included in the model, gray shading indicates variable was not included in best model as determined by AIC values, and white squares indicate variable was included in the best model and the direction of effect (or > for dichotomous variables) is provided, except for ns (non-significant). Distance to CF = distance to next continuous forest, F_IS_ = inbreeding coefficient, H_O_ = observed heterozygosity, H_E_ = expected heterozygosity, Em = *Eliurus myoxinus*, Rr = *Rattus rattus*, ANP = Ankarafantsika National Park, MCF = Mariarano Classified Forest.

	Data Set	Model	Species	Region	Sex	Forest Type	Body Mass	Forest Area	Distance to Edge	Distanceto CF	Edge Percentage
F_IS_	All	A	Em > Rr	MCF > ANP							
	All	B	Em > Rr	MCF > ANP					+		
	Fragments	C	Em > Rr	MCF > ANP							
	Fragments	D	Em > Rr	MCF > ANP					ns		
	Fragments	E	Em > Rr	MCF > ANP						ns	
H_O_	All	A	Rr > Em								
	All	B	Rr > Em								
	Fragments	C	Rr > Em					ns			
	Fragments	D	Rr > Em								
	Fragments	E	Rr > Em							ns	
H_E_	All	A	Rr > Em	MCF > ANP	ns						
	All	B	Rr > Em	MCF > ANP	ns	ns					
	Fragments	C	Rr > Em	MCF > ANP							
	Fragments	D	Rr > Em	MCF > ANP							
	Fragments	E	Rr > Em	MCF > ANP							

**Table 4:** Ecological determinants of genetic diversity—results from GAMs with each species modelled separately. Black shading indicates that variable was not included in the model, gray shading indicates that variable was not included in best model as determined by AIC values, and white squares indicate variable was included in the best model and the direction of effect (or > for dichotomous variables) is provided, except for ns (non-significant). Distance to CF = distance to next continuous forest, F_IS_ = inbreeding coefficient, H_O_ = observed heterozygosity, H_E_ = expected heterozygosity, Em = *E. myoxinus*, Rr = *R. rattus*, ANP = Ankarafantsika National Park, MCF = Mariarano Classified Forest.

	Species	Data Set	Model	Region	Sex	Forest Type	Body Mass	Forest Area	Distance to Edge	Distance to CF	Edge Percentage
F_IS_	Em	All	A	MCF > ANP							
		All	B	MCF > ANP					ns		
		Fragments	C	MCF > ANP				ns			
		Fragments	D	ns							
		Fragments	E	ns							ns
H_O_	Em	All	A	MCF > ANP							
		All	B	MCF > ANP					-		
		Fragments	C	MCF > ANP				ns			
		Fragments	D	MCF > ANP							
		Fragments	E	MCF > ANP							ns
H_O_	Em	All	A	MCF > ANP							
		All	B	MCF > ANP					ns		
		Fragments	C	MCF > ANP				ns			
		Fragments	D	MCF > ANP							
		Fragments	E								ns
F_IS_	Rr	All	A	MCF > ANP							
		All	B	MCF > ANP							
		Fragments	C	ns							
		Fragments	D	ns							
		Fragments	E	ns							
H_O_	Rr	All	A	ANP > MCF							
		All	B	ANP > MCF							
		Fragments	C								
		Fragments	D								
		Fragments	E								
H_E_	Rr	All	A	ANP > MCF							
		All	B	ANP > MCF							
		Fragments	C								

## Data Availability

All used rodent RADseq sequences are publicly available at Sequence Read Archive (NCBI) in the BioProject PRJNA993847 (Accession Numbers: SAMN36410097–SAMN36410249). Scripts used for all analyses are available from M.S.R. upon reasonable request.

## Supplementary Materials

The following supporting information can be downloaded at: https://www.mdpi.com/article/10.3390/genes14071451/s1, Table S1: Trapping Site Locations, Table S2: Individual Genetic and Geographic Data, Table S3: CV error results from ADMIXTURE Output, Table S4: Correlation Values for Analyses, Table S5: Relatedness values for all closely related dyads. Table S6: Factors influencing the abundance of four species according to model selection based on delta AICc (ΔAICc) and AICc weights.
