# Supplementary material for: Effects of Forest Fragmentation on Connectivity and Genetic Diversity in an Endemic and an Invasive Rodent in Northwestern Madagascar
Source: Genes (Basel). 2023 Jul 15;14(7):1451. doi: 10.3390/genes14071451 (PMC10378931; doi:10.3390/genes14071451)
Supplement: Supplementary file 1 [file genes-14-01451-s001.zip › Supplementary Legends.pdf]

Table S1

Study Site Locations

Name, geographic coordinates, and basic information for each study site where individuals were captured.

Table S2

Individual Genomic and Geographic Data

All samples used in final analyses with individual IDs, species, location (ANP or MCF), loci, depth, and XY coordinates.

Table S3

ADMIXTURE Output

CV error results from the ADMIXTURE program for each species on each landscape and the combined sample for populations K=1-10 used to determine the best value of K. Admixture figures are also shown for each species overall which was used to justify analyses on each location independently.

Table S4

Correlation Values for Analyses

Results of Spearman Rank correlation test between continuous predictor variables used for modeling. Significant values are in bold.

Table S5

Details on related individuals

Identity, Euclidean distance and coefficient of relatedness for all dyads with r > 0 in *E. myoxinus* and *R. rattus*

Table S6

GAM Outputs

Factors influencing the abundance of four species according to model selection based on delta AICc (ΔAICc) and AICc weights. K: the number of parameters in the model, LogLik: the maximized logarithmic likelihood, ΔAICc is the deviance of a model from the model with the lowest AICc, ω: the Akaike's

Information Criterion corrected weights. Tables are presented per variable (F_{IS}, H_O, H_E). See table 1 for input of each model (A-E).
